# Recent Discoveries in the Field of Lipid Bio-Based Ingredients for Meat Processing

**DOI:** 10.3390/molecules26010190

**Published:** 2021-01-02

**Authors:** Rubén Domínguez, Benjamin Bohrer, Paulo E. S. Munekata, Mirian Pateiro, José M. Lorenzo

**Affiliations:** 1 Centro Tecnológico de la Carne de Galicia, Rúa Galicia N° 4, Parque Tecnológico de Galicia, San Cibrao das Viñas, 32900 Ourense, Spain; rubendominguez@ceteca.net (R.D.); paulosichetti@ceteca.net (P.E.S.M.); mirianpateiro@ceteca.net (M.P.); 2 Department of Animal Sciences, The Ohio State University, Columbus, OH 43210, USA; bohrer.13@osu.edu; 3 Área de Tecnología de los Alimentos, Facultad de Ciencias de Ourense, Universidad de Vigo, 32004 Ourense, Spain

**Keywords:** healthy meat, reformulated meat products, vegetable oils, marine oils, waxes, oleogel, emulsion hydrogel, encapsulated oil, saturated fat

## Abstract

Current culture and pace of lifestyle, together with consumer demand for ready-to-eat foods, has influenced the food industry, particularly the meat sector. However, due to the important role that diet plays in human health, consumers demand safe and healthy food products. As a consequence, even foods that meet expectations for convenience and organoleptic properties must also meet expectations from a nutritional standpoint. One of the main nutritionally negative aspects of meat products is the content and composition of fat. In this sense, the meat industry has spent decades researching the best strategies for the reformulation of traditional products, without having a negative impact in technological processes or in the sensory acceptance of the final product. However, the enormous variety of meat products as well as industrial and culinary processes means that a single strategy cannot be established, despite the large volume of work carried out in this regard. Therefore, taking all the components of this complex situation into account and utilizing the large amount of scientific information that is available, this review aims to comprehensively analyze recent advances in the use of lipid bio-based materials to reformulate meat products, as well as their nutritional, technological, and sensorial implications.

## 1. Introduction

Due to the current pace and demands from a lifestyle standpoint, society tends to consume large amounts of ready-to-eat or easy-to-cook foods [1]. Many meat products can be classified as ready-to-eat (dry-ripening sausages, pâté, bologna sausage, cooked ham, etc.) or requiring minimal final preparation (burgers, frankfurters, meatballs, etc.), which make them especially attractive (and convenient) to consumers [1]. Moreover, the aforementioned categories of products have acceptable sensory characteristics and are also a valuable source of many important nutrients, including protein, B vitamins, minerals (such as iron), essential fatty acids, and amino acids [2]. However, many of these products also contain high levels of fat (mainly saturated fat) and cholesterol, and it is for this reason that the excessive consumption of these type of meat products can promote the development of several diseases, such as cardiovascular diseases and cancer [3,4,5].

Dietary guidelines from several international health organizations and federal governing agencies (such as WHO and EFSA) have recommended limiting the consumption of processed meats [4]. In fact, recent reports (WHO) indicated that the prevalence of obesity dramatically increased during the last 40 years [5] (obesity has nearly tripled since 1975), and that cardiovascular disease is the world’s main cause of death [6]. With this in mind, the international organizations (WHO and EFSA) recommend reducing total fat, as well as *trans* and saturated fatty acid (SFA) intake and increasing the consumption of monounsaturated (MUFA) or polyunsaturated fatty acids (PUFA) [7,8]. Therefore, a large part of the population is aware of the implications that diet has on health and overall wellbeing [9], so they demand low-fat and healthier products, but of course without sacrificing sensory characteristics [10].

Nonetheless, animal fat is a fundamental ingredient in processed meat products [11]. It is responsible for several important functional properties, including technological attributes (improvement in emulsion stability, influence on rheological and structural properties, regulation of the drying process in fermented/ripened meat products, etc.), sensory attributes (positive effects on texture, juiciness, color, tenderness, overall palatability, etc.), and the formation of typical and desirable aromas and flavor that contribute to the succulent and consumer-demanded characteristics of meat products (lipid-derived volatiles, lipolysis, moderate lipid oxidation, etc.) [4,12,13,14].

In order to reformulate meat products, several lipid bio-based ingredients from vegetable and marine source have been proposed in the last several years [15,16,17,18]. These lipids include healthy fatty acids (MUFA and PUFA) and also lipids that may have high amounts of natural antioxidants [19], which may have an important technological function by delaying the oxidation of the unsaturated fatty acids [20]. However, the highly unsaturated oils do not form solid structures at room temperature, which is one of the main desirable characteristics of animal fat. With this in mind, the reformulation of traditional meat products (aiming to reduce SFA and total fat amount) became a major goal for the meat industry. Therefore, in order to overcome these problems, in the last three decades several efforts were made by professionals in the industry and researchers to find viable strategies to include bio-based lipid ingredients in meat products (particularly focused on the inclusion of healthy oils with high MUFA or PUFA content and cholesterol-free) [12,21]. Multiple studies have suggested new techniques for structuring liquid oils, resulting in reformulated lipid systems with similar properties to saturated fat [2]. Consequently, the strategies proposed include the direct addition of oil (only viable in emulsified meats), the encapsulation of oils, and the addition of structured oils. This last strategy can be subdivided into two main groups, namely the use of (i) oleogels and (ii) the use of emulsion hydrogels.

The simplest way to improve the nutritional characteristic of meat products is the direct addition of a healthy oil (with or without the addition of emulsifier such as sodium caseinate [14]) to the emulsified batter. However, the technological characteristics of these meat products decrease as liquid oils are used in their formulation [22]. Moreover, the direct addition of these oils also results in a decrease in sensory properties due to the high susceptibility to oxidative degradation. To overcome some of these problems, the use of encapsulated oil was proposed. Encapsulation of oil has several benefits over direct emulsification, such as reducing lipid oxidation and masking strange or off-flavors [22,23]. Moreover, the small particles have positive effects on the texture of meat products [22]. However, the high temperatures applied during encapsulation can also cause rapid oxidation, especially when oils highly susceptible to oxidation are used (for example fish oils), and this occurs even before its application in the meat product [24]. Multiple techniques for the encapsulation of oils have been previously described, such as spray-drying, freeze-drying, complex coacervation, and external ionic gelation [22]. Although encapsulation presented some advantages, it important to highlight that similarly to direct addition of oil, the use of encapsulated oil in meat products is also limited to applications in emulsified meat products, such as frankfurters [24] or pâté [21]. However, in other meat products in which consumers desire the consistency and appearance of visible animal fat, the use of encapsulated oil is very limited because it is not capable of mimicking the appearance of fat, since it has a dry powder appearance [19]. Moreover, the encapsulation of oils requires specific and expensive equipment and is a complex, slow, and expensive technique that affects economic margins from a processor standpoint or the final price of meat products from a consumer standpoint [19]. Additionally, the application of spray-drying encapsulated oil made with arabic gum, maltodextrin, and modified starch is not recommended in cooked meat products, since it disintegrates at temperatures above 50 °C. Similarly, the microparticles produced by freeze-drying or complex coacervation can rupture at temperatures below the cooking temperatures used in meat products (about 72 °C) [22].

Although encapsulation techniques have been studied, other novel applications for the stabilization and structuring of lipid bio-based materials into gels have been proposed to replace animal fat and improve the nutritional quality of the reformulated meat products [19,25]. These strategies aim to mimic the appearance, and plastic and rheological characteristics of animal fat and at the same time improve nutritional quality and lipid profile [4]. In this regard, both, oleogels and emulsion hydrogels are two different solid oil structured systems that offer promising results as animal fat replacers in the development of meat products [26].

Recently, the use of oleogels as animal fat replacers has been proposed by several researchers [27]. Oleogels are mainly composed of oil (about of 90% of total gel) and small amounts of an organogelator that form a network allowing the conversion of liquid oil to a solid substance [4,28]. In this case, not only the oil can be considered as a lipid bio-based material, since several organogelators used in the oleogelation process are also bio-based lipids, such as waxes (carnauba wax, candelilla wax, rice bran wax, sunflower wax, beeswax, etc.), mono- and di-glycerides, and phytosterols (γ-oryzanol and *β*-sitosterol) [29,30]. Ethyl cellulose is also an important organogelator used in the reformulation of meat products [29]. However, the use of oleogel in the meat industry presents some significant disadvantages; the additional cost, and perhaps the most important, organogelators are neither classified as generally recognized as safe (GRAS) substances nor are approved for use as a bulk fat [29]. Therefore, it seems clear that one of the factors that limits the application of oleogels in the reformulation of meat products is directly linked to the approval of new regulations that allow its use [31]. Furthermore, another important concern of this strategy is the high temperatures (from 80 °C to 156 °C) during a relative longer period (from 30 min to some hours) to ensure the complete solubilization of an organogelator [28,32], which can induce lipid oxidation and limit their use in meat products. To solve this problem, the use of a natural antioxidant in the oleogelation process was proposed [32]; however, it should be noted that only lipophilic antioxidants can be added to prevent oxidation, since oleogels do not contain an aqueous phase.

The other strategy to introduce structured oils into gels is the use of emulsion hydrogels that present solid mechanical properties [13]. In this case, the gel formation process is much less aggressive to oils, as it is normally formed by non-thermal treatments. This strategy reduces the oxidative degradation of heat-labile compounds and protects bioactive compounds [4]. The formation of hydrogels involves two main steps. The first step is the production of a protein-stabilized emulsion, while the second step implies the gelation of the continuous phase [13]. Several gelling agents can be used in the development of emulsion hydrogels, including proteins (protein isolates, gelatin, etc.) and polysaccharides (alginates, agar, carrageenan, inulin, etc.). The proportion and combination of one or more of these agents result in very different hydrogel properties [4]. Other relevant advantages of emulsion hydrogels are their simplicity, lower cost, and faster production in comparison with the other strategies [3,14]. It is also important to highlight that the amount of oil incorporated in hydrogels is generally ≤50% of total gel. With this in mind, is easy to conclude that the use of hydrogels in meat products not only improves the lipid composition but also produces an effective reduction of total fat [4]. Moreover, this type of gel allows the incorporation of both lipophilic (in an oil phase) and hydrophilic (in an aqueous phase) natural antioxidants in order to limit the oxidative degradation. Similarly, some authors conclude that emulsion hydrogels are the best option to mimic hardness and water holding capacity of pork backfat [33]. Additionally, these authors also affirm that this strategy can be a better option than oleogelation to reformulate the fat in meat products.

Although several studies were conducted to improve meat products, significant problems related to the sensory properties (strange or off-flavors and odors, fishy flavors, unpleasant color, etc.) [23,30], the decrease in oxidative stability [9] due to high susceptibility of unsaturated fatty acids to autoxidation [20], and the subsequent reduction of meat product shelf-life remained. Thus, a perfect strategy to replace animal fat in processed meat products does not exist nowadays. In fact, several researchers and professionals of the food industry continue with notable research initiatives on this topic.

Therefore, taking into account the huge number of articles published during the last decades regarding the reformulation of meat products to decrease both total fat and SFA, this review focuses only on the most recent advances. It is understandable that part of the problem found in the first investigations related to this topic have already been overcome. Therefore, this review aims to analyze, in a clear and comprehensive way, the recent discoveries (from 2018 to the present) on the uses of lipid bio-based materials in the reformulation of meat products, as well as their nutritional, technological, and sensorial implications.

## 2. Sources and Compositions of Lipids

Bio-based materials are defined as commercial or industrial products that are composed of renewable biological material generated through domestic agricultural production. This includes a wide range of materials generated by plant, forestry, animal, or marine industries [34]. Conceptually, food ingredients are then defined as food-grade ingredients derived from bio-based materials [35,36]. There are many different examples of applicational uses for bio-based food ingredients. A recent book chapter written by Pandit et al. [37] classified bio-based materials into six unique categories, which were polysaccharides (e.g., plant/algal, animal-based, bacterial, and fungal), proteins (e.g., animal-derived and plant-derived), polyphenols, lipids, polyesters, and specialty polymers. These categories are applicable to bio-based ingredients as well, as there are examples of applicational uses within each category.

**Figure 1:**
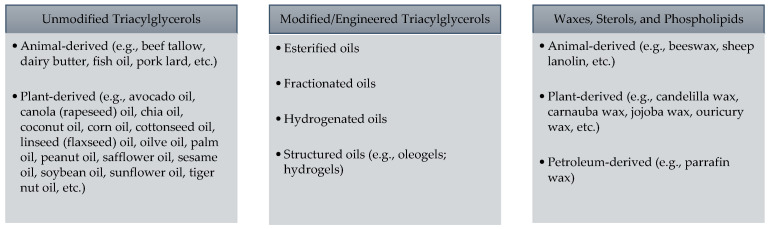
Classification of bio-based lipid food ingredients.

The focus of this review is the lipid category of bio-based ingredients and their application in meat processing. Lipid bio-based ingredients can be broken down into three major categories, which are (1) unmodified lipids, (2) modified or engineered lipids, and (3) waxes (Figure 1). With such a wide range of lipid sources within each category, there are clearly considerable differences in composition as well as environmental impacts of each lipid source. Unmodified lipids can be further categorized into those which are derived from animals and those which are derived from plants.

Generally, unmodified animal-derived lipids are higher in SFA, while plant-derived lipids are higher in MUFA and/or PUFA (Table 1). This largely determines the biological stability of lipids in food products, namely the greater rate of lipid oxidation associated with unsaturated fats [20], as well as the nutritive value of lipids in food products, namely the perceived risk of cardiovascular disease associated with consumption of saturated fats and the health benefits associated with consumption of long-chain PUFA fats [38,39,40].

There are, however, clearly exceptions to this generalization. For example, most marine-sourced lipids contain high levels of MUFA and PUFA, and some plant-sourced lipids such as coconut oil and palm oil contain high levels of SFA and low levels of unsaturated fatty acids. The content of long-chain PUFA, including linoleic acid (C18:2n-6), linolenic acid (C18:3n-3), stearidonic acid (C18:4n-3), arachidonic acid (C20:4n-6), and others have been shown to be associated with improved nutritive value and increased human health [41], reducing the incidence of cardiovascular diseases (reduces the level of cholesterol in the blood, blood pressure, etc.) and ensuring the availability of these nutrients involved in multiple biological and cellular processes. In particular, the omega-3 fatty acids eicosapentaenoic acid (EPA, C20:5n-3) and docosahexaenoic acid (DHA, C22:6n-3) are associated with improved nutritive and health benefits when consumed at levels exceeding 250–500 mg per day [42,43]. The composition of long-chain PUFA differs among unmodified lipids with marine-sourced lipids having the greatest amounts of EPA and DHA (Table 2).

A noteworthy initiative of food manufacturers is replacement of SFA with healthier unsaturated fatty acids. A challenge presented to food manufacturers when attempting to incorporate healthier lipid profiles in a food product is the different physical properties of unsaturated fats when compared with saturated fats [44,45]. In particular, the differences in thermal properties including those associated with crystallization and melting temperatures create challenges from a food processing standpoint. However, there are several lipid processing and structuring techniques that can help food processors accomplish this [44,46,47]. Such techniques include but are certainly not limited to esterification, fractionation, hydrogenation, and lipid structuring. Esterification is a term used to describe reactions that involve rearrangement of fatty acyl groups within fats or oils. Fractionation is a term used to describe the separation of fats or oils based on the solubility of higher melting triacylglycerols in the liquid transition phase.

Hydrogenation is a term used to describe the conversion of unsaturated fatty acids to either *trans* fatty acids or SFA through the addition of hydrogen to the unsaturated bonds of fatty acid chains. While these three processes have been used for a variety of different functions in foods previously, opportunities in lipid structuring may have a more promising future due to increased levels of effectiveness when compared with esterification and fractionation and increased levels of healthiness and consumer acceptability when compared with hydrogenation.

With that being stated, two options that exist for oil structuring are oleogelation and emulsion hydrogelation [4,29]. There are many different options for sourcing oils for both oleogelation and emulsion hydrogelation, many of which have been tested by researchers either independently or in combination in recent years. Highlights of these research studies are presented in the impending section of the review. Interestingly, the environmental impacts (including efficiency, land usage, deforestation, and loss of wildlife species) of sourcing these renewable (i.e., bio-based) fats/oils just now appears to be gaining traction, and this concept should continue to be considered in the future.

## 3. Incorporation of Lipid Bio-Based Ingredients into Meat Systems

The incorporation of lipid bio-based ingredients in meat products is difficult to achieve without negatively influencing technological and/or sensory characteristics. However, and despite being a great challenge for the meat industry, it should be the subject of in-depth studies in the future due to the great importance for (and opportunity to improve) consumer health within current lifestyle restraints of most consumers. Furthermore, the use of lipid bio-based ingredients also plays a fundamental role in the development of next generation agricultural industries, allowing for more environmentally sustainable and more efficient use of the planet’s resources.

With these things in mind, this section aims to present, in a comprehensive way, the most recent studies related to the application of bio-based lipid ingredients in the reformulation of meat products, as well as highlight strategies for their incorporation into meat products. Therefore, a global vision of the trend to include bio-based lipid ingredients to formulate healthy meat products exists that has relevance that reaches both the meat industry and the scientific community. The influence of this reformulation on technological, nutritional, and sensory aspects is discussed herein. Lipid inclusion strategies by emulsification or encapsulation techniques are presented in Table 3.

In recent years, as can be seen in Table 3, there have been only a limited number of studies on the use of these reformulation techniques. As for emulsification, there are only two research studies that used this strategy [14,48]. In one of these studies, the authors reformulated cooked (emulsified) lamb sausages with the inclusion of three different vegetable oils (chia oil, linseed oil, and olive oil) in the meat batter as total animal fat replacers [14]. In this case, both pork fat (control sausages) and vegetable oils (reformulated sausages) were emulsified using sodium caseinate (emulsifier). The reformulation with chia and linseed oils produced a significant reduction of fat, protein, and moisture, while olive oil did not show differences in comparison with control sausages. Regarding the nutritional benefits, it is important to highlight that all reformulated batches presented higher PUFA (chia oil and linseed oil) or MUFA (olive oil) and lower SFA content than control. Consequently, improved nutritional indices (PUFA/SFA, n-6/n-3, atherogenic index [AI], and thrombogenic index [TI]) were obtained in reformulated sausages. Despite this increase in unsaturated fats in products after processing and during refrigerated storage (90 days), only the sausages reformulated with chia oil showed a significant increase in lipid oxidation [14]. Regarding color, the values of instrumental parameters (L*, a* and b*) show researchers the color changes produced in meat products. The a* parameter is relative to the green–red opponent colors, while the b* represents the blue–yellow color. The L* represent the lightness value. In this case, the reformulation decreased redness (a*) and increased lightness (L*) in all reformulated sausages, while significant increases in yellowness (b*) were observed only in sausages reformulated with linseed oil and olive oils. These differences may be directly related to the natural color of these oils in comparison with animal fat. Additionally, the reformulation did not influence hardness (texture) of the lamb sausages. Finally, according to the sensory results, these authors found that the linseed oil showed the same consumer acceptance than control, while the lowest scores were reported for the sausages reformulated with chia oil. These results agree with another research study, which found that chia oils affect the sensory properties of reformulated meat products [14].

In the other study, the authors used *Echium* oil as a partial (50%) and a total animal fat replacer in bologna sausages [48]. In this case, the authors did not find variations in proximate or technological parameters, such as color and texture. The absence of significant differences in the composition was due to the fact that the replacement was not carried out weight by weight (animal fat by vegetable oil), but the authors actually took into account the composition of the meat, the fat, and the oil when formulating the different batches. However, a significant improvement was observed in the nutritional characteristics of reformulated sausages (higher PUFA, lower SFA, lower MUFA, and improved nutritional indices for n-6/n-3, PUFA/SFA, AI, and TI). Concerning sensory properties, total replacement of animal fat decreased the sensory quality of the sausages, while the 50% replacement showed similar sensory acceptance to the control treatment [48].

As with emulsification, there are only two recent studies that used oil encapsulation as a strategy for the reformulation of meat products [21,23]. One of them proposed the use of microencapsulated oils (chia oil, tiger nut oil, and linseed oil) to improve nutritional characteristics of deer pâté [21]. The results obtained showed that the inclusion of encapsulated oils reduced fat and increased both protein content and moisture content. From a nutritional point of view, cholesterol and SFA were reduced, whereas MUFA (with tiger nut oil) or PUFA (with chia oil and linseed oil) increased in reformulated pâtés. Moreover, chia oil and linseed oil reduced the n-6/n-3 index, while the tiger nut oil increased this index in comparison with the control. Significant differences were reported for the color parameters, where reformulation resulted in an increase of b* and a decrease of L* and also caused a reduction of texture parameters. Lipid oxidation of pâté reformulated with chia oil was significantly higher than the other batches, while tiger nut oil and linseed oil formulated pâtés presented the same values as control samples [21]. Besides this, it is important to note that all batches had oxidation values below the threshold limits for oxidation acceptability. Finally, regarding sensory properties, the pâtés reformulated with chia oil and linseed oil decreased consumer acceptability, while the acceptability of pâtés reformulated with tiger nut oil did not differ from the control samples. In contrast to these findings, reformulation of chicken (emulsified) sausages with freeze-dried and cross-linked encapsulated flaxseed oil did not affect proximate composition [23]. As occurred in pâté, the inclusion of encapsulated oil in chicken sausages decreased the hardness (texture), while the cooking loss (during sausage manufacture) also was lower in reformulated samples, mainly due to the high water-binding capacity of the agents used for the encapsulation. This parameter (cooking loss) indicates the degree of weight loss during to the cooking process, which may be due to the loss of water, fat, or a combination of both. Unfortunately, these authors did not evaluate other nutritional or sensory implications of sausages reformulation [23].

As can be seen in recent research studies, these two strategies (emulsification and encapsulation) improved the nutritional properties of reformulated meat products, even with minimal implications at the technological (with improved properties in some cases) or sensory levels. However, as mentioned before, these strategies can only be used in the reformulation of emulsified meat products, mainly due to the physical characteristics (liquid or solid) but also the visual appearance. From a visual appearance standpoint, products with visible fat cannot be reformulated with these strategies and maintain proper consistency in appearance. In this sense, the most recent investigations focused on structuring these oils into gels, which can mimic the appearance and the rheological characteristics of animal fat and can also be used in emulsified products.

One of the relevant strategies that has been increasingly studied in recent years is the structuring of oils using oleogels. In fact, from 2018 to the present, 12 papers have been published that used this type of strategy for the reformulation of meat products (Table 4).

The influence of the total replacement of animal fat in pork burgers using oleogels generated from a mixture of oils (olive oil, linseed oil, and fish oil) and two organogelators (ethyl cellulose or beeswax) was studied and presented in two different papers by the same research group [32,49]. Very similar results were obtained in both studies. In these research studies, a significant reduction of SFA and increase of MUFA and PUFA was observed. This fact resulted in an improvement of nutritional indices (n-6/n-3 and PUFA/SFA) in the reformulated burgers. Moreover, the reformulation had minimal influence on proximate composition, since the reformulation increased the fat content only in the first study [49]. In both cases, the inclusion of oleogels in pork burgers impacted textural properties by reducing shear force, while a significant increase in the L* and a* color parameters were also observed [49], which were mainly related with the natural color of both oil and organogelators, which resulted in a yellow oleogel. The authors found that the reformulated samples presented higher lipid oxidation compared with the control [49]. They related this fact to the high temperatures used for the oleogelation process and found a direct relationship between the applied temperature and oxidation (ethyl cellulose that required greater temperature also showed greater oxidation levels in the meat products). Therefore, in order to overcome this problem, the authors successfully proposed the use of curcumin as a natural antioxidant during oleogel elaboration [32]. However, the inclusion of curcumin (which presents a strong yellow–orange color) had a dramatic effect on color parameters in reformulated burgers [32]. In both cases, the use of oleogel reduced the sensory acceptability. It is important to highlight that overall acceptability of reformulated burgers with curcumin oleogels were similar to control burgers [32].

Other authors studied the effect of partial replacement of animal fat (25% and 75%) by linseed oil oleogels, using γ-oryzanol and β-sitosterol as organogelators in pork burgers [50]. In this case, the reformulation resulted in a significant and progressive decrease of moisture as well as an increase of fat content as the animal fat was replaced. Nutritionally, the replacement increased PUFA and decreased SFA and MUFA, which in turn improved the n-6/n-3 index. However, it is also important to highlight that the reformulation increased the total cholesterol of burgers. Similar to the previous studies, the inclusion of oleogels reduced hardness and increased the color parameters (L* and b*). The sensory analysis also showed that the reformulation decreased the sensory acceptability, and a preference test revealed a clear preference for the control samples (more than 80%) [50].

The use of sesame oil beeswax-based oleogels for the reformulation of beef burgers was also studied [1]. The authors found no differences in the proximate composition between the control and two animal fat replacements (25% or 50%). In contrast with the studies conducted in pork burgers, the use of oleogels decreased L* while not affecting a* or b* color parameters in beef burgers. Moreover, a significant increase in lipid oxidation and a decrease of hardness and cooking loss were observed. No information about fatty acid or cholesterol contents was reported by these authors. In this study, the reformulation of beef burgers produced a progressive increase in the overall acceptability [1].

The use of oleogels for the reformulation of sausages has also been widely studied. Thus, the total replacement of animal fat by oleogels (elaborated with rice bran wax and soybean or high-oleic soybean oil) on the quality of frankfurters [11] and bologna [51] sausages was analyzed. In both studies, the use of oleogels did not influence proximate composition, while SFA decreased and PUFA increased, which in turn improved the n-6/n-3 index. In bologna sausages, the reformulation did not affect lipid oxidation, emulsion stability, or texture parameters [51], while in frankfurters, the use of oleogels produced an increase of lipid oxidation, force (texture), and L* and b* color parameters without influencing emulsion stability [11]. In both studies, the use of this type of oleogel did not affect the sensory properties of sausages [11,51].

In another study, the authors also used oleogels for the reformulation of frankfurters [28]. However, in this case, the authors used a partial replacement (25% and 50%) of animal fat with a linseed oil beeswax-based oleogel. From a nutritional point of view, the reformulation caused an increase in fat, and specifically PUFA, and a significant reduction of moisture, protein, cholesterol, SFA, and MUFA was observed. These changes in fatty acid composition produced an improvement in nutritional indices (n-6/n-3, PUFA/SFA, AI, and TI). Regarding color, the reformulated sausages presented higher L* and b* parameters and lower a* values, while texture was not affected by the reformulation. In contrast to the sensory results reported by other studies, the reformulated frankfurters in this study showed significantly lower consumer acceptability than control samples [28].

Oleogels can also be used in other meat products beyond that of just emulsified sausages. For instance, there are two recent studies which evaluated the effect of oleogels for the reformulation of dry-fermented sausages. In one of them, the authors used linseed oil in beeswax- and phytosterols-based oleogels for the partial (20% and 40%) replacement of animal fat [27], while the other study used an olive–chia oil mixture in beeswax-based oleogels for 80% of fat replacement [26]. According to the results of proximate composition analysis, in both research studies the application of beeswax oleogels caused an increase in moisture content, while in one of these studies [26], a significant reduction of fat was observed. The use of phytosterol-based oleogels had no influence on proximate composition [27]. Regarding the nutritional characteristics, in both studies, the reformulation implied a significant reduction of SFA and MUFA and a higher content of PUFA, which produced an improvement in the most important nutritional indices. Moreover, the beeswax oleogels decreased the hardness (texture) in both research studies. Regarding color, the use beeswax oleogels increased all parameters in one study [27], while the other research study observed a significant increase of lipid oxidation and lipid-derived volatiles in the reformulated sausages [26]. Finally, a common consequence that the reformulation produced in both dry-fermented sausage products was a significant reduction in sensory acceptability [26,27].

In addition to burgers and sausages, pâté and meat batters have also been reformulated with oleogels in recent studies. The use of ethyl cellulose and beeswax oleogels with an oil mixture (olive oil, linseed oil, and fish oil) for partial (50%) and total fat replacement of pâtés did not influence proximate composition or texture [30]. In contrast, a significant decrease of SFA and MUFA and an increase of PUFA was observed, which in turn generated an improvement for the n-6/n-3 index. These authors also found a significant increase in lipid oxidation in reformulated pâtés. Regarding sensory analysis, the pâtés reformulated with the ethyl cellulose oleogels had reduced consumer acceptability, while the use of the beeswax oleogel showed similar sensory properties and consumer acceptability compared with control samples [30].

In contrast, other authors who used linseed oil in beeswax-based oleogels for the partial animal fat replacement (30 and 60%) in pâté, found that the reformulation increased moisture and decreased fat content [52]. The SFA and MUFA decreased and PUFA increased progressively with animal fat replacement, which improved the n-6/n-3 index. The reformulation also caused an increase for b* color values and a decrease for textural hardness, while a significant reduction of consumer acceptability was reported as the oleogel proportion increased [52].

The use of canola oil in ethyl cellulose oleogels for the total replacement of animal fat in meat batters were recently studied as well [53]. In this case, the authors reported that the replacement only affected (reduced) moisture content. Regarding fatty acids, a decrease of SFA and *trans* fatty acids and an increase of MUFA and PUFA were reported for reformulated samples, which in turn generated an improvement for nutritional indices (n-6/n-3 and PUFA/SFA). Moreover, the use of oleogels did not affect texture or color parameters, while decreasing lipid oxidation [53].

As a general conclusion, although there are different results depending on recent studies, there seems to be a common trend among the recent research studies that have investigated the use of oleogels to reformulate meat products. These notable points here would include an increase in lipid oxidation, change in instrumental color (especially b*), and a decrease in the sensory quality of the final product. It must be taken into account that the elaboration of all oleogels involves the application of high temperatures, which undoubtedly produces an increase in lipid oxidation of the oils (caused by high levels of unsaturated fats). To this, it must also be added that in those products where the appearance of fat is important (burgers, sausages, etc.), the consumer associates the presence of yellow “fat” with highly oxidized products. Therefore, the natural color of some oils or organogelators (e.g., beeswax) can be a disadvantage when applying them to these products. Furthermore, in general, the fat content is similar to (or even higher) than that of the control samples, which is a negative aspect from a nutritional point of view. This is because the oleogels are practically constituted by oil, while the organogelators represents a small proportion of the oleogel.

With this in mind, the most widely researched strategy to structure oils and use them to replace animal fat in meat products in recent years is the use of hydrogels (Table 5).

Burgers have been one of the most studied meat products using this strategy. In this sense, recent research on the reformulation of beef burgers (100% fat replacement) using wheat oil, algal oil, or their combination in emulsion hydrogels (alginate-based hydrogels) found a significant increase of moisture, protein, ash, and an effective reduction of total fat [19]. Regarding other nutritional properties, the reformulation increased PUFA and α-tocopherol content, while decreasing SFA and MUFA. This improved the nutritional quality of total lipid fractions, as reflected by the n-6/n-3, PUFA/SFA, AI, TI, and h/H indices. The reformulation increased textural hardness while not affecting color or cooking loss. Additionally, the reformulation process resulted in a decrease in lipid oxidation. Finally, the sensory results showed that the wheat germ oil (due to its particular flavor) reduced the sensory acceptability, while the burgers reformulated only with algal oil had similar acceptability scores compared with control samples [19].

Other researchers used the same strategy (alginate-based hydrogels) for the reformulation of deer burgers (with tiger nut oil, chia oil, or linseed oil; 100% replacement) [10] and beef burger (tiger nut oil; 50% and 100% replacement) [3]. In both cases, very similar and promising results were obtained. The results obtained in both types of burgers showed that the use of hydrogels increased the moisture, MUFA, and/or PUFA, while decreasing fat, protein, and SFA, which improved nutritional indices (n-6/n-3, PUFA/SFA, AI, TI, or h/H). This reformulation did not affect texture parameters, while decreasing cooking loss in deer and beef burgers. Color parameters showed a different trend. In deer burgers, the use of hydrogels increased a* and decreased L* values [10], while in beef burgers the reformulation increased L* and b* values [3]. Concerning lipid oxidation, the samples with hydrogels in both studies presented the lowest values, except for the chia oil treatment in deer burgers, that had significantly higher TBARS values than the other treatment [10]. In this case, and with the exception of deer burgers formulated with chia oil hydrogels (probably due to the high lipid oxidation values), the reformulation did not influence consumer acceptability in either deer and beef burgers [3,10]. Although these differences were not significant, it is important to highlight that the beef and deer burgers with 100% fat replacement with tiger nut oil hydrogels showed the highest acceptability scores.

Similarly, in another study, the authors immobilized olive oil in alginate-based hydrogels and used it for partial (33% and 66%) and total replacement of animal fat in beef burgers [61]. In this case, the reformulation increased moisture, MUFA, and PUFA and decreased fat, energy, and SFA, which improved the PUFA/SFA ratio. In burgers formulated with the hydrogels, the textural hardness and lipid oxidation rates decreased, while cooking loss increased. Finally, the scores of consumer acceptability of the reformulated beef burgers were significantly lower than control samples [61].

Other researchers studied the immobilization of a chia–linseed oil mixture (from 20% to 100% fat replacement) [62] and canola oil (100% fat replacement) [53] in polysorbate/carrageenan hydrogels, and their application in beef burgers. In both studies, the use of hydrogels caused an increase in moisture, MUFA, and/or PUFA and a decrease in total fat, SFA, and *trans* fatty acids, with the consequential improvement of nutritional indices. In the first study, the fat replacement using the chia–linseed mixture hydrogel increased hardness and lipid oxidation while reducing cooking loss and not affecting color parameters up to 60% of animal fat replacement [62]. In contrast, the total fat replacement using the canola oil hydrogels did not affect texture or color and caused a significant reduction in lipid oxidation [53]. The sensory analysis showed that the replacement of animal fat up to 60% with chia–linseed hydrogels did not affect consumer acceptability; however, the samples with a replacement of 80% and 100% presented a significant decrease in sensory quality [62].

In pork burgers, the addition of chia oil hydrogel emulsions as a fat replacer (14% and 28% replacement) caused a significant reduction in total fat and SFA and a predictable increase in PUFA content, which also improved the nutritional indices (n-6/n-3, PUFA/SFA, AI, TI, and h/H) [55]. Regarding color parameters, the reformulation increased L* and b* values and decreased a*. The hardness and cooking loss (in samples with 28% of replacement) decreased with the inclusion of hydrogels, while the lipid oxidation suffered a significant increase. The reformulation not only improved nutritional aspects of pork burgers, but also improved sensory quality and consumer acceptability [55].

A beef burger reformulated with an olive–linseed oil mixture hydrogel (50% of fat replacement) showed the opposite results (regarding proximate composition) than those observed by the previous authors [54]. In this case, the reformulation decreased moisture and increased total fat, although, in the same line of the aforementioned research studies, the reformulation improved nutritional indices due to the increase in MUFA and PUFA and the decrease in SFA. The use of hydrogels in burgers also decreased textural hardness and lipid oxidation, while increasing cooking loss and L* and b* color parameters. Sensory analysis showed that the modified burgers presented lower discoloration and the same scores for other sensory attributes in relation to control samples, which confirmed that this reformulation did not affect or improve sensory quality of beef burgers [54].

The other significant category of meat products that has been investigated for reformulation with emulsion hydrogels is sausages. In this sense, four different research articles reported results about the use of emulsion hydrogels in the reformulation of bologna sausages. The use of hydrogels containing high-oleic sunflower oil (from 25% to 100% fat replacement) [9], olive oil (100% replacement) [13], or soybean oil (50% and/or 100% fat replacement) [2,56] were proposed to modify bologna sausage formulations. These research studies reported that reformulation increased moisture and PUFA, while decreasing total fat, SFA, energy, and cholesterol contents, which improved the nutritional quality from a lipid standpoint. Two of the studies reported that the inclusion of hydrogels decreased lipid oxidation and cooking loss [9,56], while another study found a significant increase of oxidation when 100% of fat was replaced by soybean oil hydrogel [2]. The hardness (texture) of the bologna sausages with soybean hydrogels was lower than control samples [2,56]. Regarding color, generally speaking, the inclusion of hydrogels caused an increase of L* and a decrease of a* values. From these studies, only two conducted sensory analysis. In this case, the introduction of high-oleic sunflower hydrogels at inclusions levels up to 50% of fat replacement did not influence consumer acceptability [9], while the partial and total replacement of animal fat with soybean hydrogels reduced consumer acceptability. Although this result was obtained in reformulated sausages, the authors highlighted that all samples were classified as “acceptable” [56].

Similar results were reported when soybean oil hydrogels replaced total animal fat in frankfurters [33], canola oil hydrogels replaced partial (50%) or total fat in hot dog sausages [57], and camellia oil hydrogels replaced from 20% to 100% of animal fat in Harbin sausage [58]. Generally speaking, all of these studies found that the reformulation increased moisture, MUFA, and PUFA, while observing a significant reduction in total fat, energy, and SFA, which collectively caused an improvement in nutritional indices. Regarding color, all reformulations produced an increase for L*, and the use of camellia [58] and soybean [33] oil hydrogels also increased b* values. In contrast, the reformulation with canola and camellia oils reduced a* values in hot dog [57] and Harbin sausages [58], respectively. The reformulation did not influence the texture in frankfurter [33] and hot dog sausages [57], while decreasing hardness in Harbin sausages [58]. On the other hand, both partial and total replacement of fat by camellia oil decreased lipid oxidation of Harbin sausages [58], while the use of soybean oil in hot dogs did not influence oxidation [57]. Moreover, the different reformulations did not affect nor decrease cooking loss [33,57]. Finally, the total replacement of fat using soybean hydrogels reduced the consumer acceptability of frankfurters [33]. Similarly, in hot dog sausages, the partial replacement (50%) by canola oil hydrogel did not influence sensory quality, while total replacement produced a significant decrease of sensory acceptability [57].

In fresh sausages, the 90% of fat replacement with olive oil alginate-based hydrogels caused an increase of moisture and dietary fiber (the formulation included either chia flour or oat bran), and a decrease in fat, energy, and SFA, with the predictable improvement of nutritional indices [25]. Both cooking loss and hardness (texture) decreased with the application of the hydrogel, and although all samples were recognized as “acceptable”, the reformulation produced a reduction in consumer acceptability [25].

Finally, three different papers reported results about the use of hydrogels in the reformulation of dry-fermented sausages (fuet, dry-fermented sausage, and dry-fermented deer sausage). These strategies included a mixture of olive–chia oils immobilized in soy protein/gelatin hydrogel (80% of fat replacement) [26], the use of lecithin/gelatin/inulin based hydrogels with linseed oil (64% fat replacement) [59], and the application of alginate-based hydrogels with three different oils (olive oil, canola oil, or soy oil) for 50% of fat replacement in sausages [60]. The reformulation in all studies resulted in a significant increase of protein, MUFA, and/or PUFA, while increasing moisture in two out of three sausages [26,59] and decreasing this parameter in the deer sausages [60]. In all cases, the total fat and the SFA content decreased, which resulted in a significant improvement of nutritional indices (n-6/n-3 and PUFA/SFA). In fuet, the inclusion of hydrogels increased L* and b* color values [26], while in the other two sausages [59,60] the reformulation reduced these two parameters. The authors found that the use of the olive–chia oil mixture increased both lipid oxidation and lipid-derived volatiles in reformulated fuets [26]. In contrast, the use of the linseed oil hydrogel did not influence lipid oxidation during the manufacturing process (only in storage) [59], and in deer sausage similar lipid oxidation was reported between reformulated sausages and control samples [60]. Regarding texture, the use of hydrogels did not affect texture parameters in fuet [26] but decreased hardness in dry-fermented sausages [59] and increased hardness in deer sausages [60]. These discrepancies in the results are undoubtedly related to the different levels and behavior of moisture, which is the factor that influences the texture parameters of dry-fermented sausages to the greatest extent. Finally, regarding the sensory analysis results, the use of linseed oil [59] and olive–chia mixture oil [26] hydrogels produced a significant reduction of sensory quality, while the different alginate-based hydrogels (olive oil, canola oil, or soy oil) improved the consumer acceptability of dry-fermented deer sausages [60].

In general, and contrary to the reformulation of meat products with oleogels, hydrogels improved lipid profiles from a nutritional standpoint, in addition to effectively reducing the total fat content of the meat products. It can also be verified that its use reduced lipid oxidation in many cases, and that in multiple studies it did not affect (and in some cases actually improved) the sensory quality of reformulated meat products.

## 4. Conclusions and Future Trends

The use of different strategies to improve the nutritional quality of traditional meat products presents an opportunity (and a challenge) for the meat industry. Obtaining products that the consumer demands and, in turn, comply with nutritional recommendations at the international level can be a significant challenge. In this sense, the use of lipid bio-based ingredients to improve the fat fraction of meat products is one of the pillars that the industry and the scientific community have been working towards for several decades. However, and despite the enormous volume of information in this regard, there is no consensus on the best lipid engineering strategy to use, or how to apply it to meat products. Therefore, this review offers the reader the ability to review very recent information and project where trends are headed in upcoming years. At the same time, the present manuscript gives a global vision of both advantages and disadvantages as well as the limitations that each strategy has in its application for the reformulation of meat products. However, it is important to highlight that the enormous variety of meat products (fresh, cooked, dry-cured, etc.), the multiple and different processes that each product undergoes during manufacturing (chopping, cooking, drying, ripening, pasteurizing, emulsifying, etc.), as well as the different reformulation options may make it impossible to select a strategy and specific conditions for use throughout the meat industry. Even so, the main conclusions obtained from the discoveries and advances shown throughout this manuscript are listed below, as well as the considerations that future works should take into account:

- ***The type of meat product:*** It is very important to understand the type of meat product being reformulated, as well as the processes that will be carried out during its preparation, marketing, and consumption. The reformulation strategy used must be able to maintain the desired characteristics and be stable throughout the process, until the consumer finally consumes it.
- ***The type of strategy:*** There are four main strategies for the incorporation of lipid bio-based materials in meat products (emulsification, encapsulation, oleogelation, and hydrogelation). However, taking into account the differences between them, it should be noted that the best results as indicated by recent studies are obtained with the use of hydrogels. In the case of encapsulation, this is limited to be used in emulsified products in which the appearance of the fat is not important. It should also be noted that the equipment used is expensive, and that it is a complex and difficult technique to apply, which affects the final price of the product. Regarding the use of oleogels, although it is a simple technique, the main drawback is that many of the possible organogelators are not allowed to be used as substitutes for fat. Therefore, its future use relies on a change in international regulations and its inclusion for use in the reformulation of meat products. Furthermore, the intense yellow color of many organogelators (for example beeswax) imparts an undesired color to the product, while the composition of the oleogel, mainly composed of oil, does not achieve a decrease in (and in some cases even increases) the content of fat from the meat products. The oleogelation process also presents an important drawback on product quality, since it requires the application of high temperatures for long periods of time, which increases lipid oxidation and deteriorates the sensory quality of the final products. In contrast to all these aspects, the use of hydrogels is considered a cheap and simple technique that eliminates all these inconveniences. In many cases, the color obtained depends exclusively on the oil, since the emulsifiers and gelling agents used are transparent or white, which perfectly mimics animal fat. Furthermore, and taking into account that the oil content of these hydrogels is 50% or even less, the reformulated products have a reduced total fat content, while sensory quality of the reformulated products do not vary (or may even improve) in comparison with traditional products.
- ***Oil selection:*** Both individual oils and oil mixtures can be used. In this sense, it should be noted that the use of a mixture of oils (carefully selected after taking into account their composition) allows the optimization of the nutritional value and the ability to minimize negative effects (strange odors and flavors, susceptibility to oxidation, yellowish or dark colors, etc.) that each oil may have individually. It is important to highlight that although chia oil has an excellent nutritional profile, its application in the reformulations of meat products should be limited. In general, when chia oil is used (alone or in a mixture with other oils) in the reformulation of meat products, a high degree of oxidation as well as a deterioration of the sensory quality can be observed. This fact is confirmed even in studies that use the same reformulation strategy and indicate different results (with negative implications) between treatments containing chia oil and other highly unsaturated oils.
- ***Animal fat replacement:*** Finally, the percentage of animal fat replacement is also one of the key factors. Regardless of the strategy chosen, animal fat replacement levels should be carefully tested. In some products, a full replacement of the animal fat can be performed, while in other products, exceeding a specific replacement level can lead to detrimental decreases in the sensory or technological quality of the product, rendering the reformulation attempt not successful.

Therefore, as a general conclusion, all these aspects must be carefully selected and considered to achieve successful reformulation of meat products, while maintaining acceptable technological properties and sensory quality.

## Figures and Tables

**Table 1:** Composition of saturated fatty acids (SFA), monounsaturated fatty acids (MUFA), and polyunsaturated fatty acids (PUFA) for common unmodified bio-based lipid ingredients (expressed as g/100 g) and cholesterol content of animal fats (mg/100 g).

	FDC ID ^1^	SFA	MUFA	PUFA	Cholesterol
Beef tallow	171400	49.8	41.8	4.0	109
Dairy butter	790508	45.6	16.9	2.5	235
Fish (herring) oil	172340	21.3	56.6	15.6	766
Pork lard	171401	39.2	45.1	11.2	95
Lamb (subcutaneous fat)	174435	32.4	21.7	2.31	78
Avocado oil	173573	11.5	70.5	13.5	0
Canola oil	172336	7.4	63.3	28.1	0
Coconut oil	171412	82.5	6.3	1.7	0
Corn oil	748323	13.4	27.7	52.9	0
Cottonseed oil	1103859	25.9	17.8	51.9	0
Linseed (flaxseed) oil	1103860	9.0	18.4	67.8	0
Olive oil	1103861	13.8	73.0	10.5	0
Palm oil	171015	49.3	37.0	9.3	0
Peanut oil	1103862	16.9	46.2	32.0	0
Safflower oil	1103864	7.5	75.2	12.8	0
Sesame oil	1103865	14.2	39.7	41.7	0
Soybean oil	748366	14.9	22.1	57.6	0
Sunflower oil	1103867	10.3	19.5	65.7	0
Chia oil ^2^		11.1	6.7	82.2	0
Tiger nut oil ^2^		21.0	68.6	10.4	0

^1^ All values collected from the USDA FoodData Central database on 20 November 2020 (https://fdc.nal.usda.gov/), unless otherwise noted. ^2^ Values collected from Vargas-Ramella et al. [21].

**Table 2:** Composition of fatty acids for common unmodified bio-based lipid ingredients (expressed as g/100 g).

	FDC ID ^1^	C14:0	C16:0	C18:0	C16:1n-7	C18:1n-9	C18:2n-6	C18:3n-3	C18:4n-3	C20:4n-6	C20:5n-3 (EPA)	C22:5n-3 (DPA)	C22:6n-3 (DHA)
Beef tallow	171400	3.70	24.90	18.90	4.20	36.00	3.10	0.60	0.00	0.00	0.000	0.000	0.000
Dairy butter	790508	7.15	21.20	7.42	1.00	15.00	2.25	0.33	0.02	0.10	0.024	0.039	0.002
Fish (herring) oil	172340	7.19	11.70	0.82	9.64	11.96	1.15	0.76	2.31	0.29	6.273	0.619	4.206
Pork lard	171401	1.30	23.80	13.50	2.70	41.20	10.20	1.00	0.00	0.00	0.000	0.000	0.000
Lamb (subcutaneous fat)	174435	1.53	12.00	16.93	0.62	21.03	1.62	0.64	0.00	0.00	0.000	0.055	0.000
Avocado oil	173573	-	10.90	0.66	2.67	67.89	12.53	0.957	0.00	0.00	0.000	0.000	0.000
Canola oil	172336	0.00	4.30	2.09	0.21	61.74	19.01	9.14	0.00	0.00	0.000	0.000	0.000
Coconut oil	171412	16.65	8.64	2.52	0.02	6.25	1.68	0.02	0.00	0.00	0.000	0.000	0.000
Corn oil	748323	0.034	11.10	1.58	0.09	27.20	51.90	1.04	0.00	0.00	0.000	0.000	0.000
Cottonseed oil	1103859	0.80	22.70	2.30	0.80	17.00	51.50	0.20	0.00	0.10	0.000	0.000	0.000
Linseed oil	1103860	0.077	5.10	3.37	0.06	18.44	14.33	53.37	0.00	0.00	0.000	0.000	0.000
Olive oil	1103861	0.00	11.29	1.95	1.26	71.27	9.76	0.76	0.00	0.00	0.000	0.000	0.000
Palm oil	171015	1.00	43.50	4.30	0.30	36.60	9.10	0.20	0.00	0.00	0.000	0.000	0.000
Peanut oil	1103862	0.10	9.50	2.20	0.10	44.80	32.00	0.00	0.00	0.00	0.000	0.000	0.000
Safflower oil	1103864	0.00	4.86	1.92	0.10	74.84	12.72	0.10	0.00	0.00	0.000	0.000	0.000
Sesame oil	1103865	0.00	8.90	4.80	0.20	39.30	41.30	0.30	0.00	0.00	0.000	0.000	0.000
Soybean oil	748366	0.075	10.30	3.71	0.08	21.40	50.90	6.62	0.00	0.00	0.000	0.000	0.000
Sunflower oil	1103867	0.00	5.90	4.50	0.00	19.50	65.70	0.00	0.00	0.00	0.000	0.000	0.000
Chia oil ^2^		-	7.08	3.42	0.00	5.62	18.77	63.36	0.00	0.00	0.000	0.000	0.000
Tiger nut oil ^2^		-	14.03	5.59	0.00	67.16	10.15	0.21	0.00	0.00	0.000	0.000	0.000

^1^ All values collected from the USDA FoodData Central database on 20 November 2020 (https://fdc.nal.usda.gov/), unless otherwise noted. ^2^ Values collected from Vargas-Ramella et al. [21]. “-”: Data not available.

**Table 3:** Use of emulsification or encapsulation techniques for the reformulation of meat products, and their implications.

Meat Product	Microcapsule or Emulsion		Animal Fat Replacement (%)	Implications			Ref.
	Wall Material or Emulsifier	Oil		Technological and Physicochemical	Nutritional	Sensorial	
**PÂTÉ**							
Pâté	Spray-drying microencapsulated (lactose and sodium caseinate)	Tiger nut oil, chia oil, and linseed oil	50	↓ Texture parameters	↑ Moisture, protein, MUFA, PUFA	↓ Consumer acceptability. Tiger nut oil was similar to control.	[21]
				Similar lipid oxidation (except chia oil). All < for oxidized flavors.	↓ Fat, cholesterol, SFA		
				↓ L* and ↑b* color parameters	Improve nutritional indices (n-6/n-3) except tiger nut oil		
**SAUSAGE**							
Chicken sausage	Freeze-dried and cross-linked encapsulated (gelatin, gum arabic and genipin)	Flaxseed oil	-	↓ Cooking loss and texture parameters	No differences in proximate composition	-	[23]
Lamb sausage	Emulsified (sodium caseinate)	Olive oil, chia oil, and linseed oil	100	↑ L* and b* (except chia oil) and ↓ a* color parameters	↑MUFA or PUFA	↓ Consumer acceptability (except linseed oil, which had similar values to control)	[14]
				Similar texture	↓ Fat, protein and moisture (except olive oil), SFA		
				Similar lipid oxidation (except chia oil which was ↑)	Improved nutritional indices (n-6/n-3, AI, TI)		
Bologna sausage	Emulsified	*Echium* oil	50 and 100	Similar color parameters	No differences in proximate composition	↓ Consumer acceptability (except 50%, which had similar values to control)	[48]
				Similar texture			
				↑ Emulsion stability (100% replacement)	↑ PUFA and ↓ SFA and MUFA		
					Improved nutritional indices (n-6/n-3, PUFA/SFA, AI, TI)		

AI: Atherogenic index; TI: Thrombogenic index.

**Table 4:** Use of oleogelation techniques for the reformulation of meat products, and their implications.

Meat Product	Oleogel		Animal Fat Replacement (%)	Implications			Ref.
	Organogelator	Oil		Technological and Physicochemical	Nutritional	Sensorial	
**BURGERS**							
Pork burgers	Ethyl cellulose or beeswax	Olive, (44.39%), linseed (37.87%), and fish (17.74%) oil mixture	100	↓ Shear force (texture)	↑ Fat, MUFA, PUFA	↓ Consumer acceptability	[49]
				↑ Lipid oxidation	↓ SFA		
				↑ a* and b* color parameters	Improved nutritional indices (n-6/n-3, PUFA/SFA)		
	Ethyl cellulose or beeswax	Olive, (44.39%), linseed (37.87%), and fish (17.74%) oil mixture	100	↓ Shear force (texture)	No effect on proximate composition	↓ Consumer acceptability	[32]
				↑ Lipid oxidation (only with EC organogelator)	↓ SFA, ↑ MUFA, PUFA		
					Improved nutritional indices (n-6/n-3, PUFA/SFA)		
	γ-oryzanol and β-sitosterol	Linseed oil	25 and 75	↓ Hardness (texture)	↑ Fat, PUFA, cholesterol	↓ Consumer acceptability	[50]
				↑ L* and b* color parameters	↓ Moisture, SFA, MUFA		
					Improved nutritional indices (n-6/n-3)		
Beef burgers	Beeswax	Sesame oil	25 and 50	↑ Lipid oxidation	No effect on proximate composition	↑ Consumer acceptability	[1]
				↓ L* color parameter			
				↓ Hardness (texture)			
				↓ Cooking loss			
**SAUSAGES**							
Bologna sausage	Rice bran wax	Conventional or high-oleic soybean oil	100	No effect on lipid oxidation or emulsion stability	No effect on proximate composition	No effect on sensory properties	[51]
					↓ SFA, ↑ PUFA		
				No effect on texture	Improved nutritional indices (n-6/n-3)		
Frankfurter sausage	Rice bran wax	Soybean oil	100	↑ Lipid oxidation (in 10% organogelator batch) and no effect on emulsion stability	No effect on proximate composition	No effect on sensory properties	[11]
					↓ SFA, ↑ PUFA		
				↑ Force (texture)	Improved nutritional indices (n-6/n-3)		
				↑ L* and ↓ a* color parameters			
	Beeswax	Linseed oil	25 and 50	↑ L* and b* ↓ a* color parameters	↑ Fat, PUFA	↓ Consumer acceptability	[28]
					↓ Moisture, protein, cholesterol, SFA, MUFA		
				No effect on texture	Improved nutritional indices (n-6/n-3, PUFA/SFA, AI, TI, h/H)		
Dry-fermented sausage	Beeswax or γ-oryzanol and β-sitosterol	Linseed oil	20 and 40	↑ L*, b*, and a* (only in beeswax) color parameters	Small effects on proximate composition (↑ Moisture in beeswax)	↓ Consumer acceptability	[27]
					↓ SFA, MUFA, ↑ PUFA		
					Improved nutritional indices (n-6/n-3, PUFA/SFA, AI, TI)		
				No effect on texture (sterols) or ↓ hardness (beeswax)			
	Beeswax	Olive and chia oil mixture (80/20)	80	↑ L* color parameter	↑ Moisture, PUFA	↓ Consumer acceptability	[26]
				↓ Hardness (texture)	↓ Fat, MUFA, SFA		
				↑ Lipid oxidation	Improved nutritional indices (n-6/n-3, PUFA/SFA)		
				↑ Lipid-derived volatile compounds			
**PÂTÉ and MEAT BATTERS**							
Pâté	Ethyl cellulose or beeswax	Olive, (44.39%), linseed (37.87%), and fish (17.74%) oil mixture	50 and 100	↑ Lipid oxidation	No effect on proximate composition	↓ Consumer acceptability (ethyl cellulose) and no effect on sensory properties (beeswax)	[30]
					↓ SFA, MUFA, ↑ PUFA		
				No effect on texture	Improved nutritional indices (n-6/n-3)		
	Beeswax	Linseed oil	30 and 60	↑ b* color parameter	↑ Moisture, PUFA	↓ Consumer acceptability	[52]
				↓ Hardness (texture)	↓ Fat, Protein, MUFA, SFA		
					Improved nutritional indices (n-6/n-3)		
Meat batters	Ethyl cellulose	Canola oil	100	No effect on texture	↑ MUFA, PUFA	-	[53]
				↓ Lipid oxidation	↓Moisture, SFA, trans FA		
				No effect on color parameters	Improved nutritional indices (n-6/n-3, PUFA/SFA)		

**Table 5:** Use of emulsion hydrogels for the reformulation of meat products, and their implications.

Meat Product	Emulsion Hydrogel		Animal Fat Replacement (%)	Implications			Ref.
	Proportions	Oil		Technological and Physicochemical	Nutritional	Sensorial	
**BURGER AND PATTIES**							
Beef burger	Water (56%)/Prosella^®^ (6.7%; calcium sulphate, sodium alginate, wheat glucose, disodium diphosphate, sodium ascorbate)/oil (37.3%)	Wheat germ oil and/or algal oil	100	↑ Hardness (texture)	↑ Moisture, protein, Ash, PUFA, tocopherol	↓ Consumer acceptability (except algal oil with similar values to control)	[19]
				↓ Lipid oxidation	↓ Fat, energy, MUFA, SFA		
				No effect on color parameters nor cooking loss	Improved nutritional indices (n-6/n-3, PUFA/SFA, AI, TI, and h/H) (except algal oil from AI)		
	Water (56%)/Prosella^®^ (6.7%; calcium sulphate, sodium alginate, wheat glucose, disodium diphosphate, sodium ascorbate)/oil (37.3%)	Tiger nut oil	50 and 100	No effect on texture	↑ Moisture, MUFA, PUFA	No effect on consumer acceptability	[3]
				↓ Lipid oxidation and cooking loss	↓ Fat, protein, SFA		
				↑ L* and b* color parameters	Improved nutritional indices (n-6/n-3, PUFA/SFA, AI, TI, and h/H)		
Deer burger	Water (56%)/Prosella^®^ (6.7%; calcium sulphate, sodium alginate, wheat glucose, disodium diphosphate, sodium ascorbate)/oil (37.3%)	Tiger nut oil, chia oil, or linseed oil	100	↓ Cooking loss (linseed samples)	↑ Moisture, PUFA (chia and linseed), MUFA (tiger nut samples)	No effect on consumer acceptability (tiger nut and linseed oil)	[10]
				No effect on texture	↓ Fat, protein, SFA		
				↑ a* and ↓ L* color parameters	Improved n-6/n-3 nutritional index (chia oil and linseed oil samples)		
				↑ Lipid oxidation (chia oil samples)		↓ Consumer acceptability (chia oil samples)	
				↓ Lipid oxidation (tiger nut oil and linseed oil samples)			
Beef patties	Water (64%)/protein soy (10%)/oil (26%)	Olive (25%) and linseed (75%) oil mixture	50	↓ Hardness (texture)	↑ Fat, MUFA, PUFA	Improved or no effect on sensory properties.	[54]
				↓ Lipid oxidation and ↑ cooking loss	↓ Moisture, SFA		
				↑ L* and b* color parameters	Improved nutritional indices (n-6/n-3, PUFA/SFA)		
	Water (57–58.5%)/polysorbate 80 (0.05)/BHT (0.01%)/κ-carrageenan (1.5 or 3%)/oil (40%)	Canola oil	100	No effect on texture nor color	↑ Moisture, MUFA, PUFA	-	[53]
					↓ Fat, SFA, trans FA		
				↓ Lipid oxidation	Improved nutritional indices (n-6/n-3, PUFA/SFA)		
Pork burger	Water (48%)/chestnut flour (20%)/gellam gum (2%)/oil (30%)	Chia oil	14 and 28	↑ L* and b* and ↓ a* color parameters	↑ PUFA	↑ Consumer acceptability	[55]
				↓ Hardness (texture)	↓ Fat, SFA		
				↓ Cooking loss (28% replacement)	Improved nutritional indices (n-6/n-3, PUFA/SFA, AI, TI and h/H)		
				↑ Lipid oxidation			
**SAUSAGES**							
Bologna sausage	Water (37.5%)/pork skin (37.5%)/oil (25%)	High oleic sunflower oil	25, 50, 75 and 100	↓ Lipid oxidation and cooking loss	↑ Moisture	↓ Consumer acceptability (only with replacement greater than 50%)	[9]
				↑L* and b* color parameters	↓ Fat, cholesterol		
					Improved nutritional indices with replacement greater than 50% (AI, TI)		
	Water (53%)/chia mucilage (5%)/sodium alginate (0.75%)/calcium sulphate (0.75%)/sodium acid pyrophosphate (0.5%)/oil (40%)	Olive oil	100	-	↑ Moisture	-	[13]
					↓ Fat, energy		
	Water (29.5%)/soy protein isolate (4%)/inulin (16.5%)/oil (50%)	Soybean oil	50 and 100	↓ Lipid oxidation and cooking loss	↑ Moisture, PUFA	↓ Consumer acceptability (all samples considered as acceptable)	[56]
				↑ L* and ↓ a* color parameters	↓ Fat, energy, SFA		
				↓ Hardness (texture)	Improved nutritional indices (n-6/n-3, PUFA/SFA, AI, TI)		
	Water (40.75%)/soy protein isolate (2.5–5%)/chia flour (0–2.5%)/inulin (1%)/sodium tripolyphosphate (0.5%)/sodium caseinate (1%)/carrageenan (0.75%)/oil (51%)	Soybean oil	100	↑ L* and ↓ a* and b* color parameters	↑ Moisture, PUFA	-	[2]
				↑ Emulsion stability	↓ Fat, energy		
				↑ Lipid oxidation	Improved nutritional indices (n-6/n-3, PUFA/SFA, AI, TI)		
				↓ Hardness (texture)			
Frankfurter sausage	Water (29–31%)/soy protein isolate (4%)/carrageenan (0–2%)/inulin (13–16.5%)/oil (50%)	Soybean oil	100	Stable to thermal treatment	↑ Moisture, PUFA	↓ Consumer acceptability	[33]
				Similar cooking loss to control samples	↓ Fat, energy, SFA		
				No effect on texture	Improved nutritional indices (n-6/n-3, PUFA/SFA, AI, TI)		
				↑ L* and b* color parameters			
Hot dog sausages	Water (10–20%)/pork skin gel (20%)/bamboo fiber (10%)/inulin (0–10%)/oil (50%)	Canola oil	50 and 100	Similar lipid oxidation to control samples	↑ Moisture, dietary fiber, PUFA	50% replacement did not affect sensory properties	[57]
				↑ L* and ↓ a* color parameters	↓ Fat, energy, SFA		
				No effect on texture	Improved nutritional indices (n-6/n-3, PUFA/SFA, AI, TI)	100% replacement ↓ sensory properties	
				↓ Cooking loss			
Harbin sausage	Water (40%)/sodium caseinate (5.2%)/carrageenan (1.5%)/oil (53.3%)	Camelia oil	20, 40, 60, 80 and 100	↑ L* and b* and ↓ a* color parameters	↑ Moisture, MUFA	-	[58]
				↓ Hardness (texture)			
					↓ Fat, SFA, PUFA		
				↓ Lipid oxidation			
Fresh sausage	Water (58%)/chia flour or oat bran (20%)/alginate (2%)/oil (20%)	Olive oil	90	↓ Cooking loss	↑ Moisture, dietary fiber	↓ Consumer acceptability (all samples considered as acceptable)	[25]
					↓ Fat, energy, SFA		
					Improved nutritional indices (n-6/n-3 (only in chia flour), PUFA/SFA)		
				↓ Hardness (texture)			
Dry-fermented sausage (fuet)	Water (42%)/soy protein isolate (10%)/gelatin (3%)/oil (45%)	Olive and chia oil mixture (80/20)	80	↑ L* and b* color parameters	↑ Moisture, protein, PUFA	↓ Consumer acceptability	[26]
				No effect on texture	↓ Fat, SFA		
				↑ Lipid oxidation	Improved nutritional indices (n-6/n-3, PUFA/SFA)		
				↑ Lipid-derived volatile compounds			
Dry-fermented sausage	Water (50%)/soybean lecithin (3%)/pork gelatin (2%)/inulin (25%)/oil (20%)	Linseed oil	64	↑ Lipid oxidation (only during storage)	↑ Moisture, protein, PUFA	↓ Consumer acceptability	[59]
				↑ a* and ↓ b* and L* color parameters	↓ Fat, SFA		
				↓ Hardness (texture)	Improved n-6/n-3 nutritional index		
Dry-fermented deer sausage	Water (56%)/Prosella^®^ (6.7%; calcium sulphate, sodium alginate, wheat glucose, disodium diphosphate, sodium ascorbate)/oil (37.3%)	Olive, canola, and soy oil	50	Similar lipid oxidation as control	↑ Protein, MUFA, PUFA	↑ Consumer acceptability	[60]
				↓ b* and L* color parameters	↓ Moisture, fat, SFA		
				↑ Hardness (texture)	Improved n-6/n-3 nutritional index		

## Data Availability

Not applicable.
